# A wavelet-based estimator of the degrees of freedom in denoised fMRI time series for probabilistic testing of functional connectivity and brain graphs

**DOI:** 10.1016/j.neuroimage.2015.04.052

**Published:** 2016-11-15

**Authors:** Ameera X. Patel, Edward T. Bullmore

**Affiliations:** a Brain Mapping Unit, Department of Psychiatry, University of Cambridge, Cambridge, UK; b GlaxoSmithKline, ImmunoPsychiatry, Alternative Discovery & Development, Stevenage, UK; c Cambridgeshire & Peterborough NHS Foundation Trust, Cambridge, UK

**Keywords:** fMRI, Connectivity, Graph theory, Wavelet despike, Despiking, Statistic, Probabilistic, Inference, Degrees of freedom

## Abstract

Connectome mapping using techniques such as functional magnetic resonance imaging (fMRI) has become a focus of systems neuroscience. There remain many statistical challenges in analysis of functional connectivity and network architecture from BOLD fMRI multivariate time series. One key statistic for any time series is its (*effective*) degrees of freedom, *df*, which will generally be less than the number of time points (or *nominal* degrees of freedom, *N*). If we know the *df*, then probabilistic inference on other fMRI statistics, such as the correlation between two voxel or regional time series, is feasible. However, we currently lack good estimators of *df* in fMRI time series, especially after the degrees of freedom of the “raw” data have been modified substantially by denoising algorithms for head movement. Here, we used a wavelet-based method both to denoise fMRI data and to estimate the (*effective*) *df* of the denoised process. We show that seed voxel correlations corrected for locally variable *df* could be tested for false positive connectivity with better control over Type I error and greater specificity of anatomical mapping than probabilistic connectivity maps using the nominal degrees of freedom. We also show that wavelet despiked statistics can be used to estimate all pairwise correlations between a set of regional nodes, assign a *P* value to each edge, and then iteratively add edges to the graph in order of increasing *P*. These probabilistically thresholded graphs are likely more robust to regional variation in head movement effects than comparable graphs constructed by thresholding correlations. Finally, we show that time-windowed estimates of *df* can be used for probabilistic connectivity testing or dynamic network analysis so that apparent changes in the functional connectome are appropriately corrected for the effects of transient noise bursts. Wavelet despiking is both an algorithm for fMRI time series denoising and an estimator of the (*effective*) *df* of denoised fMRI time series. Accurate estimation of *df* offers many potential advantages for probabilistically thresholding functional connectivity and network statistics tested in the context of spatially variant and non-stationary noise. Code for wavelet despiking, seed correlational testing and probabilistic graph construction is freely available to download as part of the BrainWavelet Toolbox at www.brainwavelet.org.

## Highlights

• We lack good estimators of degrees of freedom (*df*) in denoised fMRI time series.
• Wavelet despiking can simultaneously denoise and estimate *effective df* post-denoising.
• We show Type I error controlled single-subject probabilistic inference for seed connectivity.
• We describe new methods for building and probabilistically thresholding brain graphs.
• We extend these methods to probabilistic inference for time-varying connectomics.

## 1 Introduction

Connectome mapping has become a major focus of neuroscience research in the last few years. Functional mapping techniques, such as functional MRI (fMRI), are among the most commonly used tools for investigating the network architecture of the brain. Yet we are currently somewhat limited in our ability to use probabilistic reasoning to test estimates of functional connectivity – such as the correlation between two fMRI times series – against an appropriate null hypothesis with good control over Type I error rates. One obvious reason why probabilistic inference is difficult in the context of fMRI connectivity and network analysis is that the “raw” multivariate time series data have a complex distribution that is spatially anisotropic or heterogeneous. A functional MRI time series is not “white”, meaning that the time points are independent of each other, it is “colored”, meaning that low frequencies or positive autocorrelations exist and the time points are not independent of each other (Weisskoff et al., 1993, Friston et al., 1994, Friston et al., 1995, Boynton et al., 1996, Bullmore et al., 1996, Zarahn et al., 1997). A corollary of the non-white nature of fMRI time series is that the *effective* degrees of freedom (*df*) will generally be less than the *nominal* degrees of freedom or simply the number of time points in the series, *N*.

It is now beyond doubt that a large proportion of the variance and covariance of a resting state fMRI run is not generated by blood oxygenation level dependent (BOLD) mechanisms, and does not reliably represent neurovascular activity. All raw data must therefore be extensively pre-processed to eliminate, as far as possible, non-specific sources of variance such as respiration and head movement. In particular, recent developments in pre-processing have focused on methods to achieve control over the pernicious effects on connectivity estimators of even a few brief bursts of low amplitude head movement (so-called micro-movements ~ 0.1 mm). The denoising pipelines for resting-state fMRI that have evolved over the last 5 years have been progressive in recognizing and controlling the effects of micro-movements. But all of these denoising methods necessarily imply some loss of the degrees of freedom in the time series. For example, if the “raw” time series had *N* = 100 nominal degrees of freedom and this was reduced to *N* = 68 by “scrubbing” or censoring of micro-movement-contaminated time points, then the (*effective*) *df* of the denoised time series is not immediately known but it is expected to be less than 68.

It is potentially important to know the *df* of a time series because if we know *df*, then we can do parametric inference on other simple time series statistics. For example, we can test the null hypothesis that the correlation coefficient *r* between two voxels is zero by a transformation of *r* to a standard Normal *Z* score that involves *df* as a factor. If we don't know *df*, we can't use Fisher's *r*-to-*Z* transform for valid inference on correlations between fMRI time series. Absent a better estimate of *df*, we might assume that *df* = *N*: but then hypothesis testing based on simple parametric statistics like the Fisher *r*-to-*Z* transform will be biased and there will be (many) more false positive tests than would be expected given the probability of Type I error, e.g. *P* = 0.05, and the number of tests conducted, or search volume.

A good general strategy for estimating *df* is to somehow whiten or decorrelate the pre-processed fMRI time series. In a previous paper (Patel et al., 2014), we described a new wavelet-based method for denoising motion artifacts from resting-state fMRI time series, which we called “wavelet despiking”. This data-driven method characterizes motion artifacts as large non-stationary events across multiple frequencies, using the prior assumption that the underlying signal of interest is more stationary than the artifacts, and removes them in an unsupervised and spatially adaptive way. Here we demonstrate that this same algorithm can be used to estimate the *df* of the time series at each voxel, generating *df* maps for individual subjects, based on the level of denoising required at each voxel. For each subject, the algorithm takes into account the number of *df* that were lost at each voxel in order to remove the large non-stationary events, and generates an estimate of the *df* at each voxel, for each wavelet scale (frequency band). We demonstrate that use of these spatially-variable *df* maps provides effective Type I error control for single-subject seed connectivity analysis, thus validating this approach for single-subject statistical inference. In addition, we generalize this method to graph theoretic analysis, and introduce a new method for generating and building graphs by assigning *P* values to edges, still keeping the traditional method of weighting edges by correlation (or another measure of functional connectivity), but building the graphs based on the probabilistic properties of each edge. Applying wavelet despiking to fMRI data thus has two distinct advantages over other approaches: first, it provides effective motion artifact removal (Patel et al., 2014), and secondly, it enables robust estimation of the voxel-wise *df* remaining after removal of such artifacts, thus enabling robust single-subject statistical inference.

We accompany this article with a new release of the BrainWavelet Toolbox to include tools for generating single-subject *df* and statistical maps for seed-based and graph theoretic analysis, which is freely available to download from www.brainwavelet.org.

## 2 Materials and methods

### 2.1 Subjects and fMRI data acquisition

**Fig. 1:**
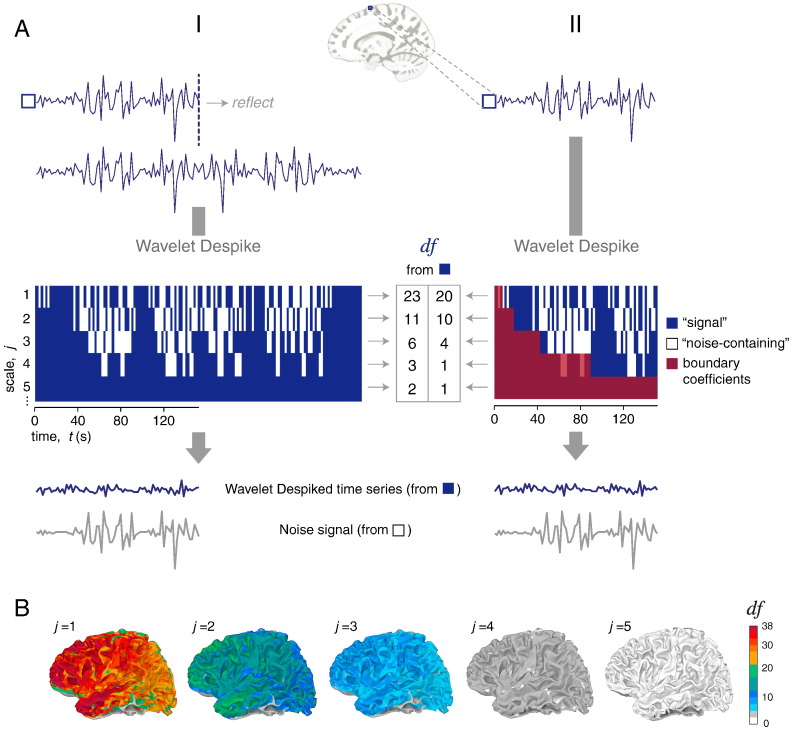
A wavelet-based estimator of the (*effective*) degrees of freedom, *df*, in functional MRI time series. Each voxel time series is processed using the wavelet despiking algorithm. The first step in this algorithm is computing the maximal overlap discrete wavelet transform (MODWT) of the time series. (A) Column (I) shows the MODWT computed using the *reflection* boundary, and (II) shows the same for the *periodic*, or *circular* boundary. This step converts the time series into a matrix of scale (*j*) vs. time (*t*). Denoising is conducted on this matrix to produce a final binary matrix indicating coefficients affected by non-stationary events (“noise” coefficients ${\tilde{W}}_{\phi ,j,t}$ shown in white) and unaffected coefficients (“signal” coefficients ${\tilde{W}}_{\alpha ,j,t}$ shown in blue). For more details on the method, see Section 2.3 and Patel et al. (2014). The *df* are estimated from the “signal” coefficients as described in Eqs. (5), (6), for each wavelet scale (frequency band) taking advantage of the fact that the MODWT is an approximate band-pass filter with pass-bands 1/2^*j* + 1^ < |*f*| ≤ 1/2^*j*^. If the *periodic* boundary condition is used, the boundary coefficients (shown in red, column II) need to be discounted in order to obtain an unbiased estimate of the *df*, thus resulting a reduction in the *df* available at each scale (as described in Eq. (6)). The number of boundary coefficients at each scale is dependent on the wavelet filter used (see Eq. (4)); here we used the Daubechies L = 8 wavelet. (B) Spatial *df* maps for each wavelet scale (*j*), in a randomly selected subject, after the voxel-wise *df* had been estimated as in panel A, column I.

**Fig. 2:**
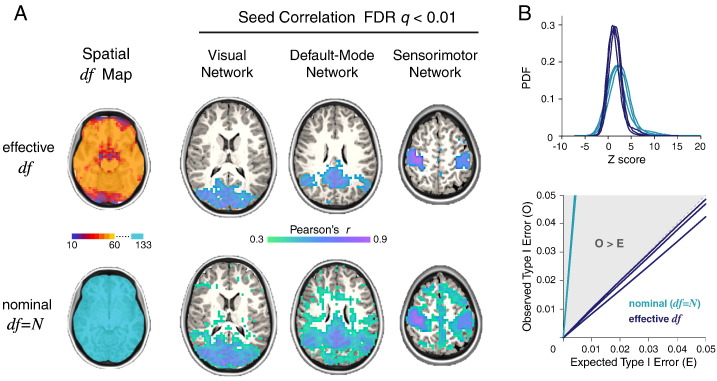
Single-subject seed correlation analysis of functional connectivity using probabilistic thresholds. For this set of analyses, we compared the *effective* degrees of freedom (*df*) produced by the wavelet despiking algorithm (see Fig. 1 and Section 2.4 of the Methods), with the *nominal* degrees of freedom, (assuming *df* = *N*) or the number of time points (a commonly used estimate of *df*). The subject's EPI image was fully pre-processed and “band-pass” filtered using the MODWT, retaining detail in scales 2–4, representing a commonly analyzed frequency range: 0.02 < *f* < 0.13 Hz. The *df* estimate at each voxel was produced by adding the *df* estimates across scales 2–4. (A) The far left column shows the *df* maps used by both methods. Seed correlation maps were thresholded at a *P* value equivalent to FDR *q* < 0.01. Significant correlations are shown in the figure, along with their corresponding weights (*r*), for the wavelet-based *df* estimator (upper) and for nominal *df* = *N* (lower). (B) The upper panel shows the distribution of *Z* scores in the three networks shown in panel A, with the wavelet-based *df* estimator (dark blue) compared to assuming nominal *df* = *N* (light blue). The lower panel shows results of a series of permutations tests designed to estimate the observed vs. expected Type I error for the three seed correlation results in panel A. For any given *P* value, the observed Type I error (*y*-axis) should not exceed *P* (*x*-axis). The wavelet-based *df* estimator (dark blue) provided good Type I error control, whereas assuming nominal *df* = *N* (light blue) did not.

**Fig. 3:**
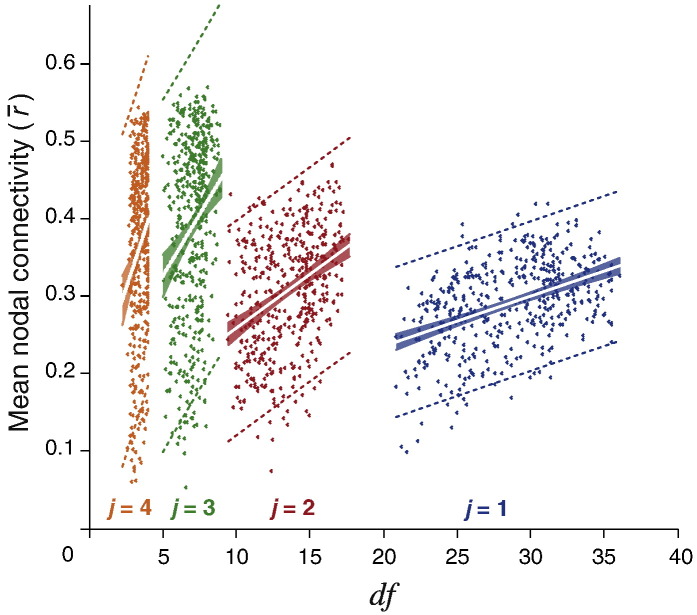
Mean nodal connectivity shows strong linear dependence on the *df*. Voxel-wise time series were parcellated into 470 regions and a connectivity matrix was generated for each wavelet scale (frequency band) by computing the Pearson correlation *r* on MODWT wavelet coefficients, between each pairwise combination of nodes. The mean nodal connectivity represents the average of all non-zero correlations between a given node and all other nodes in the brain. The voxel-wise *df* estimate was generated for each wavelet scale, by wavelet despiking. The nodal *df* were computed by averaging the *df* of all voxels in each region (defined by the 470 region parcellation template), at each scale. This resulted in an *df* estimate for each of 470 regions at different wavelet scales. The mean nodal connectivity ($\bar{r}$) was then plotted against the nodal *df* for the highest four wavelet scales (*j*), representing the most commonly analyzed frequency bands: *j* = 1, 0.13 < *f* < 0.25 Hz; *j* = 2, 0.06 < *f* < 0.13 Hz; *j* = 3, 0.03 < *f* < 0.06 Hz; *j* = 4, 0.02 < *f* < 0.03 Hz. The mean nodal connectivity showed strong linear dependence on the *df* at all four wavelet scales (*P* < 0.0001, F test).

To illustrate our new methods for statistical inference, we used 3 cohorts. Cohort 1 is a previously published cohort of 22 children (Power et al., 2012) with an average age of 8.5 years. All subjects gave assent with parental consent as approved by the Washington University Human Studies Committee. The data were obtained from Washington University at St. Louis and the surrounding areas. Scans were acquired on a Siemens MAGNETOM Tim Trio 3.0 T scanner. Each dataset comprises a T1-weighted MPRAGE structural image (TE = 3.06 ms, TR partition = 2.4 s, TI = 1000 ms, flip angle = 8°) with a voxel resolution of 1.0 × 1.0 × 1.0 mm, and a BOLD functional image, acquired using a whole-brain gradient echo echo-planar (EPI) sequence with interleaved slice acquisition (TR = 2.2–2.5 s, TE = 27 ms, flip angle = 90°), and with voxel dimensions of 4.0 × 4.0 × 4.0 mm. This cohort contains many subjects with high spatial variability in artifacts, caused by head movement, and was thus used for analyses in Fig. 1, Fig. 2, Fig. 3, Fig. 5.

Cohorts 2 and 3 (Lynall et al., 2010) are a group of 13 normal adults (average age 33.3 years) and a group of 11 people diagnosed with schizophrenia (average age 32.8 years) as defined by DSM IV (American Psychiatric Association, 2000). All subjects provided written informed consent as approved by the Addenbrooke's NHS Trust Local Research Ethics Committee. The data were collected on a General Electric Signa 1.5 T scanner at the BUPA Lea Hospital (Cambridge, UK). Each dataset comprises a T1-weighted MPRAGE structural image with a voxel resolution of 1.0 × 1.0 × 1.0 mm, and a run of 512 gradient-echo T2^⁎^-weighted echo-planar BOLD functional images (TR = 2 s, TE = 40 s, flip angle = 70°). The functional images have voxel dimensions of 3.05 × 3.05 × 7.00 mm. Subjects from cohort 2 and 3 were used to demonstrate the methods presented in Fig. 6, Fig. 7. These cohorts comprise runs of a more typical length, the number of scans is consistent across subjects, and many subjects in cohort 3 contain high temporal variability in the presence of artifact and were thus used to demonstrate the sliding-window methodology in Fig. 7.

### 2.2 Functional image pre-processing

Functional and structural images were processed as in our previous paper (Patel et al., 2014), using AFNI (Cox, 1996) and FSL (Smith et al., 2004) software, and the BrainWavelet Toolbox for denoising motion artifacts (www.brainwavelet.org, Patel et al. (2014)). Pre-processing was divided into two main modules: Core Image Processing, and Denoising.

Core Image Processing included the following steps: (i) slice acquisition correction; (ii) rigid-body head movement correction to the first frame of data; (iii) obliquity transform to the structural image; (iv) affine co-registration to the skull-stripped structural image using a gray matter mask; (v) standard space transform to the MNI152 template in MNI space; (v) spatial smoothing (6 mm full width at half maximum); (vi) a within-run intensity normalization to a whole-brain median of 1000. All spatial transforms were applied in one step to avoid incremental blurring of the data that can occur from multiple independent transforms.

Denoising steps included: (vii) wavelet despiking (performed voxel-wise with the BrainWavelet Toolbox); (viii) confound signal regression including the 6 motion parameters estimated in (ii), their first order temporal derivatives, and ventricular cerebrospinal fluid (CSF) signal; and (ix) a wavelet “band-pass” filter. In this last step, the Maximal Overlap Discrete Wavelet Transform (MODWT) was used to produce a set of scales (frequency bands), and coefficients from scales representing frequency bands of interest were recomposed to produce frequency-filtered time series.

### 2.3 Wavelet despiking

This algorithm was designed to remove both high and low frequency non-stationary events related to subject movement, at a voxel level, in the wavelet domain (Patel et al., 2014). The key steps in the algorithm's operation are outlined below.

For each voxel, the wavelet despiking algorithm first computes the MOWDT of the time series using Mallat's pyramid algorithm (Mallat, 1998). We refer to the wavelet transform of each voxel time series *X_t_* as ${\tilde{W}}_{X,j,t}$, where *j* represents the scale, or frequency band, *t* represents time; and where *j* = {1, …, *J*}, *t* = {0, …, *N* − 1}, *J* is the number of scales, and *N* is the number of time points. We defined *J* conservatively as the largest positive integer satisfying the condition:

(1) $$J\leq log_{2}\left(\frac{N}{L-1}+1\right)$$

where *J* ∈ ℤ^+^ and *L* is the filter length, in this case *L* = 4.

For the MODWT, we used a fourth-order Daubechies wavelet filter (L = 8), which is an asymmetric filter that has been found to provide good decorrelation of wavelet detail coefficients for the Discrete Wavelet Transform (DWT, Bullmore et al. (2001); Laird et al. (2004)), such that the coefficients are approximately independent. In addition, to avoid the biasing effects of discontinuities arising from the time series boundaries, we used a *reflection* boundary which yielded 2 *N* × *J* wavelet coefficients. Use of a *periodic* (or *circular*) boundary condition will yield fewer *df* for an unbiased estimate due to boundary effects. This is discussed further below. The only disadvantage of using the *reflection* boundary is an increase in computation time and RAM usage (Percival and Walden, 2006), which we do not find to be a limiting factor with current computational power. The redundancy of the MODWT is key to the denoising features of the wavelet despiking algorithm. This is because the MODWT can isolate transient non-stationary phenomena with high temporal precision in all frequencies, with minimal impact on neighboring coefficients.

The wavelet despiking algorithm then identifies coefficients contaminated by large non-stationary phenomena (artifacts) as maxima and minima chains and separates the wavelet detail coefficients ${\tilde{W}}_{X,j,t}$ for each voxel into two disjoint sets representing those part of maxima and minima chains (“noise” coefficients, ${\tilde{W}}_{\phi ,j,t}$), and “signal” coefficients $\left({\tilde{W}}_{\alpha ,j,t}\right)$. These two sets are additive in the wavelet domain.

So, for each voxel,

(2) $${\tilde{W}}_{X,j,t}={\tilde{W}}_{\alpha ,j,t}+{\tilde{W}}_{\phi ,j,t}\text{.}$$

For further details on the methods used to identify these two sets of coefficients, please see Patel et al. (2014). In the final step of the algorithm, each time series is recomposed from the “signal” coefficients (${\tilde{W}}_{\alpha ,j,t}$) only, using the inverse MODWT (iMODWT) and the inverse pyramid algorithm (Mallat, 1998), to yield a denoised time series.

### 2.4 Effective degrees of freedom (*df*) estimation

The *effective* degrees of freedom (*df*) are estimated during the wavelet despiking step. The MODWT is a modified version of the DWT with many similar properties, but it is a highly redundant non-orthogonal transform yielding *N* coefficients at each wavelet scale. The redundancy allows non-stationary events to be located in any scale with high temporal precision, and removed efficiently with minimal impact on neighboring coefficients or time points, in addition to allowing natural definition for any time series length. Both of these factors make the transform particularly useful for denoising fMRI time series. However, these properties also mean that estimation of the *df* at each scale is not as trivial as for the DWT, where the *df* could be simply estimated as the number of large detail coefficients.

There are a number of well-established methods for computing the *df* from the MODWT (Percival and Walden, 2006). If we wish to make no assumptions about the shape of the Spectral Density Function, and want to avoid overestimating the *df* for short runs (simulations in Percival (1995) suggest that *N* < 128 is small enough), the simplest, and most conservative, estimate of the *df* is the most appropriate (Percival and Walden (2006), pp. 314). This is the method we implement. This method takes advantage of the fact that the MODWT is an approximate band-pass filter with pass-bands 1/2^*j* + 1^ < |*f*| ≤ 1/2^*j*^. In this case, the *df*$\left(\hat{\eta }\right)$ would be defined as follows:

(3) $${\hat{\eta }}_{j}=\max\left\{\left\lfloor \frac{M_{j}}{2^{j}}\right\rfloor ,1\right\}$$

where *M_j_* refers to the number of non-boundary coefficients at scale *j*. For the *reflection* boundary, *M_j_* = *N_j_* = *N*, where *N_j_* is simply the number of coefficients at scale *j*. For the *periodic* (or *circular*) boundary condition, the number of boundary coefficients are a factor of the filter length, so for each scale *j*,

(4) $$M_{j}=N_{j}-\min\left\{\left(2^{j}-1\right)\left(L-1\right),N\right\}\text{.}$$

We extend the definition of $\hat{\eta }$ stated in Eq. (3) above (Percival and Walden, 2006) to accommodate the denoising performed by the wavelet despiking algorithm. That is, we simply subtract all noise coefficients $\left({\tilde{W}}_{\phi ,j,t}\right)$ that were removed during the wavelet despiking process in the estimation of *df*. Thus, for the *reflection* boundary (as we use in this article), we redefine the *df*$\left(\hat{\eta }\right)$ after wavelet despiking as:

(5) $${\hat{\eta }}_{j}=\max\left\{\left\lfloor \frac{N_{j}-N_{\phi ,j}}{2^{j}}\right\rfloor ,1\right\}$$

and for the *periodic* (or *circular*) boundary condition:

(6) $${\hat{\eta }}_{j}=\max\left\{\left\lfloor \frac{M_{j}-M_{\phi ,j}}{2^{j}}\right\rfloor ,1\right\}$$

where *M*_*ϕ*,*j*_ refers to the number of noise coefficients *N*_*ϕ*,*j*_ not in the range of boundary coefficients, i.e. ∉ ${\tilde{W}}_{X,j,t}$ for *t* = 0, …, min{*L*_*j*_ − 2, *N* − 1}.

Assuming approximate independence across scales, the *df* can be combined across scales using an additive model and the total *df* across scales will, by definition, be ≤ *N*.

### 2.5 Dynamic window length estimation

For dynamic window length estimation, we first computed the fraction of brain voxels containing a signal coefficient (${\tilde{W}}_{\alpha ,j,t}$, taking account of all wavelet scales) at each time point *t*. We call this the signal fraction (*SF*).

(7) $$SF_{t}=\frac{1}{n\left(V\right)\cdot J}\cdot \sum_{{\tilde{W}}_{j,t}\in V}\left(\sum_{j=1}^{J}{\tilde{W}}_{j,t}\right)$$

where *V* is the set of all brain voxels.

For a specified target “*effective*” window length (*w*), the dynamic window length *D* starting at time *t* was defined as:

(8) $$D_{t}=\sum_{i=t}^{T}SF_{i}\text{,}$$

where *T* is the largest integer in the range {*t* + 1, …, *N*} allowing *D_t_* to be in the range {*w* − 1, *w*}.

### 2.6 Micro-movement and transient noise diagnostics

We used two diagnostics for assessing the level of micro head movements and transient noise present in each dataset. The first was the framewise displacement (*FD*, Power et al. (2012)), which is the sum of the absolute derivatives of the 6 head movement parameters (*x*, *y*, *z*, *α*, *β*, *γ*), representing 3 planes of translation and 3 planes of rotation. Rotational parameters (yaw *α*, pitch *β* and roll *γ*) were converted to distances by computing the arc length displacement on the surface of a sphere with radius 50 mm (as in Power et al. (2012)). *FD* at time *t* = 0 was given the value 0 in order for the length of *FD* to equal *N*. For *t* = {1, …, *N* − 1}, where *N* = the number of time points,

(9) $$\begin{array}{l} FD_{t}=\sum_{d\in D}|d_{\left(t-1\right)}-d_{t}|+50\cdot \frac{\pi }{180}\cdot \sum_{r\in R}\left|r_{\left(t-1\right)}-r_{t}\right|\\ \mathrm{where}\;D=\left\{x,y,z\right\}\;\&\;R=\left\{\alpha ,\beta ,\gamma \right\}\text{.} \end{array}$$

The second diagnostic, the spike percentage (*SP*, Patel et al. (2014)), was used to assess the level of artifact removal required in each dataset. For any given time point, *SP_t_* was defined as the percentage of voxels containing a noise coefficient in wavelet scale 1 $\left({\tilde{W}}_{\phi ,1,t}\right)$ in that frame of data. For a run of *N* time points, the *SP* is therefore a vector of *N* points. So, for *t* = {0, …, *N* − 1},

(10) $$SP_{t}=\frac{100}{n\left(V\right)}\cdot \sum_{{\tilde{W}}_{\phi ,1,t}\in V}{\tilde{W}}_{\phi ,1,t}$$

where *V* is the number of voxels.

### 2.7 Parcellation and graph analysis

For graph analysis, voxels were down-sampled into 470 approximately same-sized regions. The template was a randomly subparcellated in-house version of a fusion atlas created based on the Harvard–Oxford cortical and subcortical atlas (probabilistically thresholded at 25%) in MNI space, and the Oxford thalamic connectivity atlas (Behrens et al., 2003). Region sizes were constrained such that no region was more than twice the size of any other region, and no regions crossed anatomical boundaries or hemispheres defined by these atlases. This parcellation template has been made free for download at www.brainwavelet.org. Time series within these regions were averaged and the coordinates expressed as centroids.

For regional estimates of *df* based on the *df* maps generated by the wavelet despiking algorithm, the same parcellation template and averaging method were used to down-sample the voxel-wise *df* maps. We add that it is possible for the voxel-wise estimates of *df* to be treated in the same way as the time series themselves, so if time series weighting is desired (e.g. to account for gray matter probability of voxels) during estimation of regional time series, then the same method can be applied to the *df* maps. This also applies to functional parcellations, in other words, any functional parcellation template used to parcellate the fMRI time series can be used to parcellate the *df* maps.

After definition of regional time series, graph analysis was performed in R using iGraph v.0.6.5.2 (http://cran.r-project.org/web/packages/igraph/).

### 2.8 Statistical tests

Statistical tests were performed either at a voxel level using the voxel-wise *df* maps produced during wavelet despiking (for seed connectivity analysis), or on parcellated *df* maps (for graph analysis). In each case, the number of *df* maps produced was equal to *J*, or the number of scales (see Eq. (1)). Pearson correlation of time series was then either estimated in the wavelet domain for each scale separately, e.g. for graph analysis, (and then analyzed in combination with *df* maps produced for each corresponding wavelet scale), or in the time domain. The latter was used for analysis of wide frequency ranges spanning multiple scales, such as for the seed correlation analysis (Fig. 2). In this case, the MODWT was used to “band-pass” the time series, retaining information from the desired scales, and the recomposed time series were correlated in the time domain. The *df* corresponding to each time series were combined using an additive model across the corresponding scales.

The resulting correlation matrices or seed correlation *r* maps were then converted to test-statistic *Z* scores using Fisher's *r*-to-*Z* transform, correcting for the edge or pair-wise *df*. So, for each correlation *r* at scale *j*,

(11) $$Z_{j}=0.5\cdot \ln\left(\frac{1+r_{j}}{1-r_{j}}\right)\cdot \sqrt{{\hat{\eta }}_{j}-3}\text{.}$$

We refer to the test-statistic *Z* score, which is the *r*-to-*Z* transformed correlations (0.5 ⋅ ln(1 + *r*_*j*_/1 − *r*_*j*_)) divided by the standard error $\left(1/\sqrt{{\hat{\eta }}_{j}-3}\right)$, simply as the *Z* score.

These *Z* scores could then be compared to the standard Normal distribution and converted to *P* values. This yielded a *P* value map for each correlation matrix (*P* values assigned to edges) or seed correlation map, which could then be thresholded probabilistically. *P* value thresholds were adjusted according to the Benjamini–Hochberg procedure for controlling the false discovery rate (FDR, Benjamini and Hochberg (1995); Benjamini and Yekutieli (2001); Genovese et al. (2002)). The threshold was determined as the largest *P* value *P_i_* (where *i* represents the voxel, or edge, index after all *P* values have been sorted from smallest to largest) such that,

(12) $$\begin{array}{lll} P_{i}\leq \frac{i}{N}\cdot \frac{q}{c\left(N\right)}, & \mathrm{where} & c\left(N\right)=\sum_{i=1}^{N}\frac{1}{i} \end{array}$$

where *N* is the number of observations, *q* is the desired FDR limit, and *c*(*N*) is a constant determined by the harmonic sum of the number of observations. This definition of *c*(*N*) was used as it makes no assumptions about the joint distribution of *P* values (Genovese et al., 2002) and results in a harsher threshold cut-off. However, it may be relatively safe to assume a Gaussian distribution of *P*, giving *c*(*N*) = 1 (Genovese et al., 2002).

### 2.9 Permutation testing

To test Type I error rates at a single-subject level, time series were randomized to create a null model, while preserving certain basic properties of the underlying signal. Under these circumstances the number of significant observations as a fraction of the total observations at any given *P* value should not exceed *P* (the probability of Type I error) in order for the statistical test to be meaningful (Bullmore et al., 1999). Here, time series were scrambled in the Fourier domain to randomize the phase using white noise, but preserve amplitude information (Theiler et al., 1992). We note that it is possible to randomize fMRI time series in the wavelet domain by temporal resampling of wavelet coefficients (Bullmore et al., 2001, Bullmore et al., 2004, Breakspear et al., 2003, Breakspear et al., 2004, Sendur et al., 2007), a process known as “wavestrapping”, and that this may produce lower false positive rates than Fourier resampling (Laird et al., 2004). Wavestrapping has been well characterized for the DWT (Percival et al., 2000) and applied to fMRI data in the time domain (Bullmore et al., 2001), and spatiotemporal domain (Breakspear et al., 2003, Patel et al., 2006), but this transform has time series length restrictions. Translating this procedure to redundant transforms such as the MODWT requires further analysis in fMRI data, and may require block resampling of wavelet coefficients, which is beyond the scope of this article.

## 3 Results

### 3.1 Single-subject probabilistic seed correlation analysis

We began by analyzing seed correlation maps for a single subject. In order to threshold these maps by a FDR-adjusted *P* value, we first needed an estimate of the degrees of freedom. We compared two estimates: the *effective* degrees of freedom (*df*) generated by the wavelet despiking algorithm (see Fig. 1 and Section 2.4 of the Methods), and the *nominal* degrees of freedom (*df* = *N*), simply the number of time points. As the wavelet despiking algorithm treats each voxel independently, it generates an estimate of the *df* at each voxel and at each scale (see Fig. 1B), appropriately reducing the *df* in voxels where harsher denoising is required.

The MODWT was used to “band-pass” the time series retaining information in scales 2–4, which represents a commonly analyzed frequency range of 0.02 < *f* < 0.13 Hz. To obtain an estimate of the *df* at each voxel in this frequency range, we combined the *df* estimated at each voxel across scales 2–4 using an additive model, to generate one number per voxel, representing the *df* at that voxel in the frequency range 0.02 < *f* < 0.13 Hz (see Fig. 2A, far left column). The *nominal* degrees of freedom across all voxels (assuming *df* = *N*) was simply 133, or the number of time points.

We chose 3 seeds (2 × 2 × 3 voxels) each located to elicit a well-defined resting-state network: (the visual network, the default-mode network, and the sensorimotor network), and computed the seed correlation (*r*) maps, using Pearson correlation, between these seeds and all other voxels in the brain. We defined the *df* for the correlation *r* at each voxel as the minimum *df* between the seed and the voxel to which it was correlated. The *r* map was then transformed to a *Z* map by applying Fisher's *r*-to-*Z* transform, corrected for the *df* of each correlation (see Eq. (11)). To compare to the results of assuming that *df* = *N* (i.e. the nominal degrees of freedom), we also estimated *Z* maps assuming that *df* = 133 for all voxels. These voxel-wise *Z* values could then be simply converted to *P* values by comparison with the standard Normal distribution. The *P* value threshold corresponding to FDR *q* < 0.01 was computed using the Benjamini–Hochberg procedure (Benjamini and Hochberg, 1995) where the constant *c*(*N*) (Eq. (12)) was determined by the harmonic sum of the number of voxels (see Section 2.8 of the Methods for more information). The probabilistically thresholded *r* maps, at *q* < 0.01, are shown in Fig. 2A.

The wavelet-based estimator of *df* appeared to produce substantially fewer spurious correlations to voxels not known to be associated with these networks. The probability distribution of *Z* scores associated with the voxel-wise *r* values in the three networks analyzed can be found in Fig. 2B (upper panel). As the method accounts for spatial variability in *df*, different *P* values were assigned to the same *r* value according to the number of *df* remaining after denoising.

In order to quantify the false positive rate for hypothesis testing predicated on the wavelet-based estimator of *df*, we conducted a series of permutation tests. For each set of seed correlations, we randomized the seed and voxel time series using Fourier-based resampling, to estimate the correlation coefficient between all pairs of randomly permuted time series under the null hypothesis that the true correlation is zero (see Section 2.9 of the Methods, Theiler et al. (1992)). As before, we estimated the seed correlation *r* maps and converted these to *P* value maps corrected for locally variable *df* ≤ *N*, and estimated the false positive rate of the test (*α*) as the number of significant voxels defined by an arbitrary critical *P* value, as a fraction of the total search volume or number of voxels tested (Bullmore et al., 1999). This process of time series permutation in the Fourier domain followed by probabilistic hypothesis testing over a range of critical *P* thresholds was repeated 100 times to assess the robustness of the results. For any given nominal *P* value, the observed false positive rate *α* should not exceed *P*. If the observed false positive rate exceeds the nominal size of the test *P*, then the test is invalid; if *α* is less than or equal to *P* then the test is valid and increasingly conservative as *α* becomes smaller than *P*. As shown in Fig. 2B (lower panel), the wavelet-based method provided good Type I error control where the observed Type I error *α* was less than the expected Type I error for all three seeds. Assuming *df* = *N* (nominal degrees of freedom) resulted in very poor Type I error control with observed error rates in excess of those expected, for all three seeds. This means that the Normal theory based hypothesis testing of *Z*-transformed correlation coefficients is not valid on the assumption the *effective df* is simply the number of time points available. Because the *effective df* is typically much smaller than the nominal degrees of freedom (*N*), significance tests assuming that *df* = *N* will lead to an excessive and uncontrolled number of false positive results.

### 3.2 Probabilistic thresholding of functional connectivity matrices to construct brain graphs

Graph theoretical analysis of fMRI data typically involves the following key steps. After voxel time series have been down-sampled (parcellated) into regional time series *n*, a statistical measure of functional connectivity is estimated between all pairwise regional time series, resulting in an *n* × *n* association matrix or functional connectivity matrix. The pairwise correlations are typically then individually thresholded so that small correlations are set to zero and larger correlations that exceed the arbitrary threshold are represented as edges between regional nodes in an adjacency matrix or brain graph. Thresholds are usually specified to result in graphs of variable connection density, where connection density is the number of non-zero edges divided by the total number of possible edges, (*M* × *M* − *M*) / 2. As there has been no simple method for assigning *P* values to correlations between fMRI time series it has been difficult to use probabilistic thresholds so that graphs are specified in terms of an overall or family-wise false positive error rate as well as, or instead of, a connection density.

One might assume that simply adding edges in decreasing order of the strength of positive correlation would at least imply that edges were added to the graph in increasing order of their probability under the null hypothesis. But, this is only true if the *df* is consistent across all voxels or monotonically decreases with decreasing correlation. However, as the presence and magnitude of artifacts are spatially inhomogeneous (Patel et al., 2014, Satterthwaite et al., 2013, Wilke, 2012), and therefore removal of these artifacts requires spatially-variable denoising (Patel et al., 2014), this assumption does not necessarily hold. Indeed, after wavelet despiking, the mean connectivity in regions that have been more harshly denoised due to the presence of movement (and other large non-stationary) artifacts is much lower, as one would predict (see Fig. 3). In this figure, the mean nodal connectivity represents the average Pearson correlation (*r*) between a node and all other nodes in the network (defined by the 470 region parcellation template, see Section 2.7 of the Methods for more details), computed in the wavelet domain, using the MODWT, for each wavelet scale (or frequency band). The *df* of each node is the mean *effective df* of all voxels in the region. As demonstrated by this analysis, the mean regional connectivity shows strong linear dependence on the *df* across the four commonly analyzed wavelet scales. This is the side effect of denoising artifacts; put simply, the more denoising required in a region (lower *df*), the less signal will remain, and therefore, the expected connectivity of that region will be lower.

**Fig. 4:**
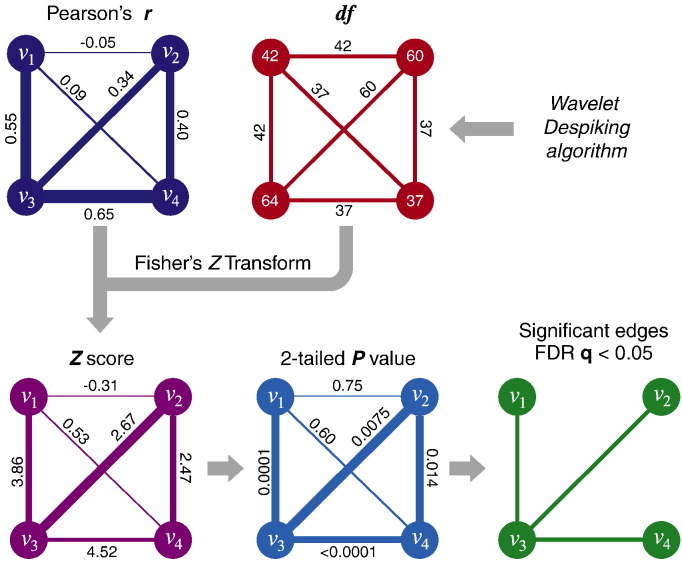
Schematic of probabilistic thresholding for construction of graphs from a functional connectivity matrix. This method comprises the following five steps. (1) Defining edge connectivity: the connectivity (here, Pearson correlation) between each set of nodes (*v*_1_,..., *v*_4_) representing fMRI time series is used to weight each possible edge. (2) Edge degrees of freedom: voxel-wise *df* maps generated by wavelet despiking are parcellated by averaging voxel-wise *df* within each region (see Fig. 5A, right panel for an example). If a gray matter weighting is applied during time series parcellation, this same weighting can be applied to the regional *df* calculation. The *df* of the edge connecting two nodes is then assigned the minimum *df* of the pair of nodes. (3) *r*-to-*Z* transform of edge weights: the measures of functional connectivity (edge weights) are then converted to *Z* scores by Fisher's *r*-to-*Z* transform (Eq. (11)) accounting for edge *df* estimated in step 2. (4) Assigning *P* values to edges: *Z* scores can then be converted to 2-tailed *P* values by comparison to the standard Normal distribution. (5) Probabilistic thresholding of edges: *P* values can be thresholded to define edges. In this example, we thresholded by a *P* value corresponding to FDR *q* < 0.05 using the Benjamini–Hochberg procedure (Benjamini and Hochberg, 1995, Benjamini and Yekutieli, 2001), where the number of observations was the total number of edges. Edges can be unweighted (identically unit weight) or weighted by the pairwise correlation to form binary or weighted graphs. In this example, the three significant edges do not exactly correspond to the three edges with the highest functional connectivity after taking account of the *df* at each node.

To address these issues, we generalize the wavelet-based method for estimating *df* and hypothesis testing of pairwise correlations to the application of constructing a graph from a correlation matrix (Fig. 4). As described in the figure legend, this method involves 5 key steps: (1) obtaining estimates of edge connectivity, such as Pearson correlation; (2) using the *df* map to produce edge *df*; (3) performing an *r*-to-*Z* transform of edge weights; (4) assigning *P* values to edges; and (5) probabilistically thresholding edges after adjusting for multiple comparisons.

### 3.3 Topological properties of probabilistically thresholded graphs

**Fig. 5:**
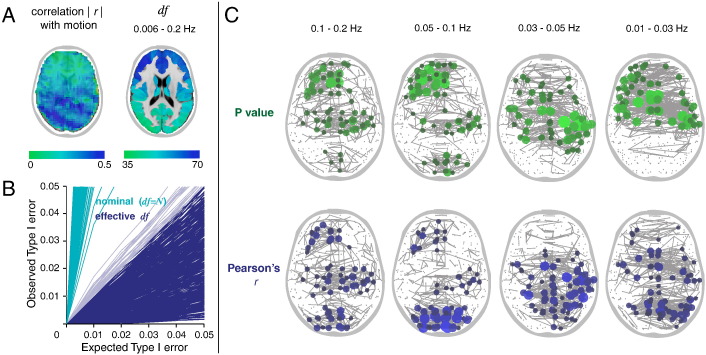
Graphs constructed by probabilistic thresholding and correlation thresholding. (A) Left panel shows the correlation between framewise displacement (see Section 2.6 of the Methods) and voxel-wise time series for an fMRI dataset acquired with high levels of head movement. Dark blue areas show high artifact regions. Right panel shows parcellated *df* maps for the subject after time series denoising by wavelet despiking. (B) Results of a series of permutations tests designed to evaluate our method for estimating nodal *df*. Observed vs. expected Type I error rates for each node are shown in dark blue for the wavelet-based *df* estimator, and in light blue for the equivalent analysis assuming nominal *df* = *N*. (C) The top 0.5% of edges ranked by *P* value (shown in green) vs. the top 0.5% of edges ranked by Pearson correlation *r* (shown in blue) plotted between their corresponding coordinates in the brain. The node size is proportional to node degree at this connection density.

We next analyzed the impact of accounting for the *df* of edges on the location of the strongest edges. Here we analyzed a subject that exhibited high levels of rotational head movement, and thus required harsher denoising by the wavelet despiking algorithm in posterior regions of the brain. We show the non-spatially-homogeneous distribution of artifacts by correlating the framewise displacement (see Section 2.6 of the Methods) with voxel-wise time series (Fig. 5A, left panel). As posterior regions of the brain had to be despiked more harshly, these regions had fewer *df*. Fig. 5A (right panel) shows the total *df* remaining, across all wavelet scales (non-Nyquist frequencies), at each node. Nodal *df* were computed by averaging voxel-wise *df* estimates. We evaluated whether this method of averaging voxel-wise *df* to produce nodal *df* provided good Type I error control by use of permutation testing (see Section 2.9 of the Methods). For each node (to be used as a seed), we permuted its corresponding time series using Fourier resampling (see Section 2.9 of the Methods), and the time series of all other nodes in the brain. Next, using the edge *df* (the minimum *df* between the pair of connecting nodes) we estimated the number of significant connections at a range of *P* values (expected Type I error rates, see Fig. 5B). This was repeated 100 times for each node, and the observed Type I error was calculated as the average of the observed error in each of the 100 trials. This was repeated for all 470 nodes, resulting in a total of 47,000 permutations. As we demonstrate in Fig. 5B, the nodal *df* estimation, based on the wavelet estimator of *effective df*, provided good Type I error control, whereas the equivalent analysis using *nominal* degrees of freedom (assuming *df* = *N*) did not.

For this individual subject, we next took the *P* value matrix and identified the top 0.5% of edges (the edges with the lowest *P* values), and plotted these edges topographically (Fig. 5C). The same was done for the correlation matrix *r*, taking the top 0.5% of edges to be those with the highest Pearson correlation, and we compared the results obtained by the two methods (see Fig. 5C). As shown in this panel, the two methods produced somewhat different topologies across the four most commonly analyzed wavelet scales (*j* = 1, 0.13–0.25 Hz; *j* = 2, 0.06–0.13 Hz; *j* = 3, 0.03–0.06 Hz; and *j* = 4, 0.02–0.03 Hz). The difference was most marked in posterior regions of the brain where the loss of signal due to the presence of artifacts (which required harsher denoising to remove) resulted in a significant loss of *df*, and thus higher *P* values. This then resulted in a change in the spatial distribution of the strongest edges and a change in which nodes had the highest degree.

**Fig. 6:**
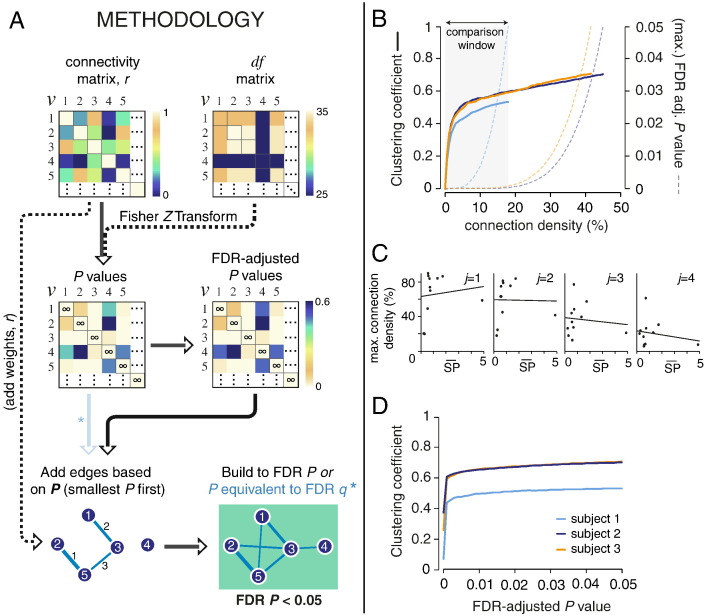
Proposed methodology for building graphs, and thresholding connection density, based on the statistical properties of edges. (A) Toy example demonstrating the proposed methodology. Connectivity matrices (*r*) are transformed to *Z* scores, accounting for edge *df* (here shown in matrix form), and subsequently to *P* values by comparison with the standard Normal distribution. These *P* values can then be adjusted for multiple comparisons. Edges can then be added to the growing network based on their *P* value, smallest *P* first, and built up to a given *P* value threshold. Here we use a FDR-adjusted *P* value threshold of 0.05. (B) Demonstration of the approach shown in A for the clustering coefficient. The clustering coefficient was calculated after incremental addition of edges by *P* value, and plotted against the connection density. This was demonstrated for 3 subjects in cohort 2 with differing levels of artifact – high (orange), medium (dark blue) and low (light blue) – as estimated by the mean spike percentage (see Section 2.6 of the Methods). Graphs were built up to a FDR-adjusted *P* value of 0.05. The maximum *P* value across all edges in the network for each connection density is shown by the correspondingly colored dotted line. Cross-subject comparison can be conducted within the “comparison window” indicated. (C) The mean spike percentage plotted against the maximum connection density possible if the network is thresholded at FDR-adjusted *P* < 0.05, at wavelet scales 1–4 (*j* = 1, 0.13 < *f* < 0.25 Hz; *j* = 2, 0.06 < *f* < 0.13 Hz; *j* = 3, 0.03 < *f* < 0.06 Hz; *j* = 4, 0.02 < *f* < 0.03 Hz). (D) The clustering coefficient values in panel (B) re-plotted as a function of *P* value, demonstrating an alternate approach for cross-subject comparison of network measures.

We also evaluated probabilistic thresholding for graph construction in a dataset of healthy adult subjects (cohort 2), for a commonly calculated graph measure, the clustering coefficient. For illustrative purposes, we show the results at wavelet scale *j* = 2 (0.06–0.13 Hz), as this is a scale in which group differences have commonly been reported (as previously demonstrated for this cohort, Lynall et al. (2010)). For each subject, edges were ranked according to their *P* value, and added incrementally starting with the smallest *P*. After each addition, the clustering coefficient was calculated and plotted against the connection density (see Fig. 6B, upper panel). As shown in this figure, the cut-off connection density can vary significantly between subjects. For each subject, this *P* value cut-off in theory depends on the intrinsic connectivity, and the level of denoising required. As we show in Fig. 6B, the maximum connection density does not appear to depend on the level of denoising in this cohort, as the subject with the highest *df* had the lowest connection density cut-off. We analyzed this further by regressing the maximum connection density (for a maximum network FDR-adjusted *P* value of 0.05), against the level of denoising required for each subject (the mean spike percentage) in this cohort, for wavelet scales 1 to 4 (spanning the frequency range of 0.02–0.13 Hz). The linear fit was not predictive of the data (at *P* = 0.05, F test), suggesting no significant linear dependence of the maximum connection density on the level of denoising in this cohort (Fig. 6C).

### 3.4 Probabilistic thresholding for construction of time-varying graphs

Finally, we explored how the wavelet-based estimator of *df* could be further generalized to analyze the temporal dynamics of networks through use of sliding window approaches to time series analysis. One of the limitations in our interpretation of such information (i.e. of non-stationary BOLD fluctuations) from resting-state time series is that artifacts manifest as large non-stationary events (Patel et al., 2014) and we have no information about the temporal location of changes in endogenous BOLD fluctuations. Furthermore, as the presence of artifacts can vary throughout time, harsher denoising may be required at some time points than others, resulting in a non-uniform distribution of *df* throughout time. One of the problems then with using a fixed window is that time series within windows that have been more harshly denoised will have fewer *df* and, as a result, lower detection power. Here, we address this limitation by demonstrating that window lengths can be dynamically adjusted according to the *df* within specific temporal windows, in order to prevent fluctuations in *df* between windows.

**Fig. 7:**
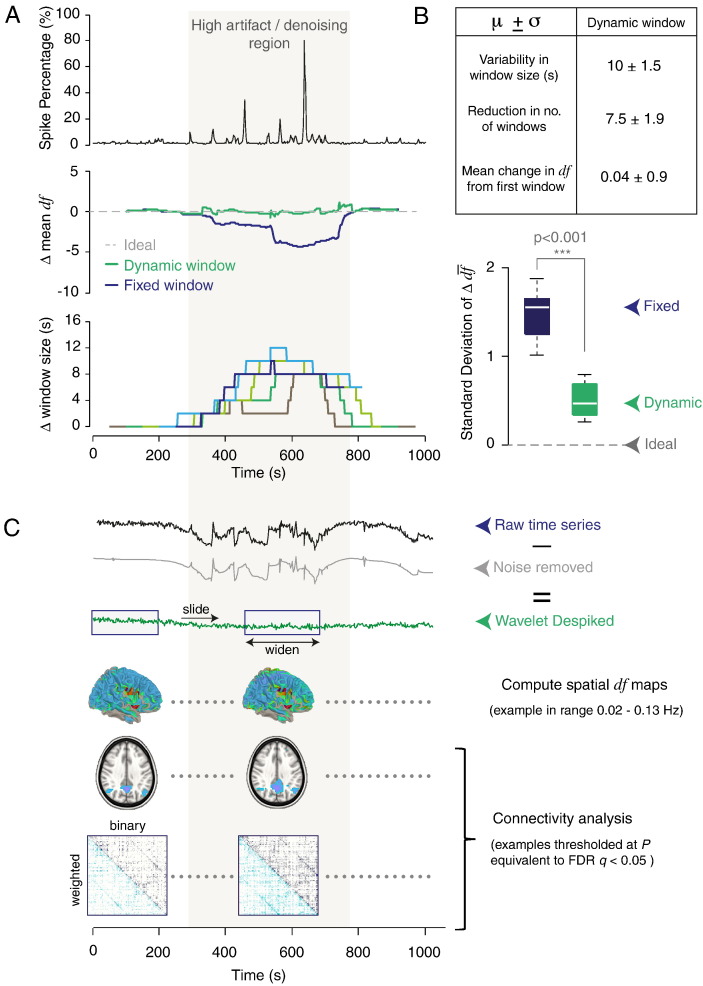
Application of the *df* method to time-windowed analysis. (A) Upper panel shows the spike percentage (indicating the level of wavelet despiking required at each time point) for an example subject from cohort 3 showing high artifact levels in the middle of the run. The middle panel shows the mean (across voxels) change in total within-window *df*, from the first window. The blue line represents the fixed window method where a sliding window of length 200 s was used. The green line represents the same for the dynamic window approach. In this case, the window length was dynamically increased (minimum window size of 200 s) in low *df* regions in order to minimize window-to-window variation in *df*. Points plotted indicate the middle of each window. The lower panel shows the change in window size through time using the dynamic window approach, for window lengths of 100, 200, 300, 400 and 500 s. Points plotted indicate the middle of each window. (B) The table describes the properties of the set of dynamic windows calculated for a range of minimum window lengths between 100 and 500 s. Boxplots show the standard deviation of the change in *df* from the first frame of data (e.g. of the trace shown in the middle of panel A), for a range of different (minimum) window lengths. (C) Example of how the dynamic window approach can be used to generate windowed spatial *df* maps, which can then be used for functional connectivity or graph analyses, and statistically thresholded as described in Fig. 2, Fig. 4, Fig. 6.

We demonstrate application of this method to a patient from cohort 3 with high levels of artifact in the middle of the run. Use of a fixed length window results in a drop in mean *df* at high artifact time points occurring in the middle of the run (Fig. 7A, middle panel, demonstrated for a window length of 200 s), whereas use of a dynamic window (detailed in Section 2.5 of the Methods) which increases in length upon reaching high artifact (or low *df*) time points, minimizes variation in windowed *df* through time. Examples of how the window length changes using this dynamic approach, can be found in Fig. 7A (lower panel) for window lengths of 100, 200, 300, 400 and 500 s. We quantified the effectiveness of the dynamic window approach at reducing window-to-window variability in *df* by measuring the standard deviation of the change in mean *df* through time (Fig. 7A, middle panel) for a range of window lengths spanning 100 to 500 s. The variability in *df* was significantly lower compared to the fixed window length approach (*P* < 0.001, 1-tailed T test, Fig. 7B boxplots). In absolute terms, window-to-window variation in *df* did not exceed 1 *df* using the dynamic approach, at a cost of on average 7.5 fewer windows and on average 10 s difference in window length between the shortest and longest windows (Fig. 7B, table).

After computing dynamic window lengths to minimize temporal variability in *df*, spatial *df* maps can be generated for each window, and the time series can be analyzed in the same way as described in the sections above. After probabilistic thresholding of connectivity maps or matrices, results from windows can be matched or compared by *P* value (as described above), given that variations in *df* across windows have been minimized.

## 4 Discussion

One of the limitations in the statistical interpretability of fMRI data at a single-subject level is the estimation of *effective* degrees of freedom (*df*). Here we present a method for estimating *df* at a voxel level, using wavelets, which uniquely results in the production of spatially-variable *df* maps for individual subjects. This method, based on the wavelet despiking algorithm we have previously described for the removal of large non-stationary artifacts (Patel et al., 2014), has the additional advantage of being able to account for signal and *df* loss as a result of artifacts (and artifact denoising) present variably in space and time.

### 4.1 Type I error controlled single-subject statistical inference for functional connectivity

Statistical inference at a single-subject level requires both robust artifact denoising and a method for estimating the *df*. As artifacts in fMRI data are not spatially homogeneous and can be present in both low and high frequencies, good removal requires spatially-adaptive denoising that can operate in multiple frequencies (Patel et al., 2014). However, the presence of spatially-variable artifacts means that some areas of the brain will unavoidably have less signal. For estimating the *df*, there exist a number of whitening methods that can be used to decorrelate fMRI data in order to maximize statistical independence. These include Independent Component Analysis (ICA) and Discrete Wavelet Transforms (DWTs, Percival and Walden (2006)), as well as Fourier-based methods (Worsley and Friston, 1995). Where the dimensionality is constrained a priori, ICA runs the risk of biasing the *df* estimate, particularly if components are selected by the operator. In addition, many of the most commonly used spatial ICA algorithms in fMRI, such as InfoMax and FastICA (implemented in FSL, Smith et al. (2004)), may optimize signal sparsity, not independence (Daubechies et al., 2009). Such methods have the added limitation that they provide one estimate of the *df* for each subject. For example, if a subject were scanned twice, and in the second run exhibited a higher level of movement, one would expect the signal-to-noise ratio and *df* to be lower in the second run. However, if the movement primarily affected anterior or posterior regions of the brain, as is common in fMRI data (Wilke, 2012, Satterthwaite et al., 2013), one would not expect the loss of *df* to be spatially homogeneous, just as we would not observe the motion artifacts to be spread in a spatially homogeneous manner (Patel et al., 2014). Whereas ICA may result in a reduced estimate of the *df* for the subject's second run, wavelet transforms can be used to produce locally reduced estimates of the *df*, by estimating the *df* at a voxel level, thus affording the additional advantage of accommodating spatial variability in *df*.

In a previous paper (Patel et al., 2014), we described a method for data-driven and spatially-adaptive denoising of artifacts from multiple frequencies, which we showed to be highly effective at removing motion artifacts. Here, we used the same algorithm to generate spatial *df* maps for single subjects which combines the whitening properties of discrete wavelet transforms with the method for time series denoising (see Fig. 1A and Section 2.4 of the Methods). This analysis method results in localized denoising in voxels (and frequencies) in which the artifacts occur, and corresponding localized estimation of *df*. This method has the further advantage of being able to characterize voxel-wise *df* in individual wavelet scales (frequency bands), by taking account of the innate *df* present in a given frequency and the level of denoising required in that particular frequency to remove artifacts (see Fig. 1B for an example of spatial *df* maps for a single subject).

Once the *df* are known, converting a single seed connectivity (*r*) map into a *P* value map for probabilistic thresholding is then simple. The functional connectivity between a seed and a given voxel can be assigned the minimum *df* between the seed region and the voxel, and the *df* estimate can inform conversion of *r* maps to *Z* maps using Fisher's *r*-to-*Z* transform. Voxel-wise *Z* scores can then be compared to the standard Normal distribution and assigned *P* values. These *P* values can then be corrected for multiple comparisons, for example, as we show, using an FDR correction. As we show in Fig. 2, this method for estimating the *df* results in excellent Type I error control.

### 4.2 A statistically-reasoned methodology for graph theoretic analysis

Next, given the voxel-wise *df*, there is a natural application to graph theoretic analysis. Here, we propose a new methodology for building graphs based on the probabilistic properties of edges (see Fig. 4), which confers a number of advantages and addresses some important issues in graph theoretical analysis of fMRI data.

#### 4.2.1 A probabilistically-informed order of adding edges

Traditionally when building graphs, edges are ranked by functional connectivity or weight, and added serially starting with the highest connectivity edge. However, if the time series of some of the nodes connected to these “high connectivity” edges had been denoised heavily, these time series would have lower *df*, which should be reflected in their edge strength. Indeed, as we show in Fig. 3, the mean connectivity of a node relates strongly to the level of denoising, or the *df* of that node. Put another way, a high artifact node will require harsher denoising, and as a result will have less signal (represented by lower connectivity) and fewer *df*. Thus, without any consideration of the *df*, one would not be able to differentiate between lower connectivity due to more denoising (as a result of more artifacts present in those regions), and truly (neurobiologically) attenuated functional connectivity. Conversely, regions of similarly high connectivity cannot be compared on a level plane if they both have different *df*. In order to take account of nodal *df* when building graphs, we propose that edges be added in accordance with their *P* value (as binary edges or weighted by their measure of functional connectivity), lowest *P* value first. As we demonstrate in Fig. 5C, this can affect the network topology, as nodes with more denoising (and resulting lower *df*) will have weaker edges than they would if the local *df* had not been considered. This method should also reduce spurious connectivity to high artifact regions, by penalizing their connectivity with low *df*.

#### 4.2.2 Identifying the maximum interpretable connection density

One of the challenges of estimating the network properties of the brain is in identifying which connection density to estimate these properties at. Typically graphs are built at a range of connection densities, and properties such as the clustering coefficient, modularity, and efficiency are calculated at each of these densities. Subjects are then matched by connection density and group comparison is performed across a range of densities. In the absence of a method defining a maximum connection density, this method often results in comparison of networks with a substantial number of “noisy” edges, which could then bias the graph measures. Here we propose that the maximum connection density of a given brain network should be identified by thresholding the graph by incremental addition of edges in order of increasing *P* value, up to a critical *P* value ≤ 0.05, corrected for the multiple comparisons entailed by individually testing a large number of edges for statistical significance. For example, the connectivity matrix *r* can be converted to an FDR-adjusted *P* value matrix, and graphs can then be constructed with strong control over Type I error that a false positive edge has been added to the graph. These probabilistically thresholded graphs can be analyzed as binary graphs (all edges = 1), or each edge can be weighted by the functional connectivity between the connected pair of nodes (Fig. 6A). If the measure of functional connectivity between paired nodes contains both positive and negative values, the edges of these thresholded graphs can have both positive and negative weights, as *Z* scores can be converted to 2-tailed *P* values (Fig. 4). “Noisy” edges with either positive or negative weights will be excluded during the probabilistic thresholding process.

#### 4.2.3 Group comparison of graph measures across subjects

For group comparison after probabilistic thresholding of connection densities, there are two potential approaches. The first is a version of the traditional method where subjects are matched by connection density. As all subject networks will have been built to different maximum densities, group comparison can then only be conducted up to the largest connection density covered by all subjects in the cohort. In our example in Fig. 6B, this would be up to 17.9%. However, if the level of denoising does not relate strongly to the connection density cut-off, as we show for cohort 2 (Fig. 6C), an alternative approach can be used where graph measures are compared across subjects by matching *P* values instead of connection density (Fig. 6D). This may be appropriate if differences in connection density cut-off relate more to differences in functional connectivity, or if comparison is to being made across two cohorts with large global differences in functional connectivity.

### 4.3 Application to time-varying connectomics

Finally, we demonstrate how our method can be extended to the analysis of temporal network dynamics. Sliding window approaches for MEG and resting-state fMRI data, which attempt to analyze non-stationary fluctuations in network organization through time, have become increasingly popular (Chang and Glover, 2010, Bassett et al., 2011, Kitzbichler et al., 2011). However, interpretation of results from such analyses is challenging. One of the main limitations is that artifacts in resting-state time series often manifest as large non-stationary events (Patel et al., 2014) which may diminish or destroy any underlying non-stationary BOLD fluctuations. In addition, we have no information about the temporal location of endogenous fluctuations. Reliable interpretation of such information thus relies on good denoising strategies. To further complicate matters, the presence of artifacts can vary substantially throughout time, meaning that harsher denoising will be required at some time points than others, resulting in a non-uniform distribution of *df* through time. As we discuss above, the presence of artifacts (and harsher denoising) will result in less signal and lower detection power of networks at those time points. If this is not accounted for, then observed fluctuations in network results may in fact be related to lower detection power at that point in time, due to harsher denoising. In order to apply the statistical methods we describe above and compare information across windows, the *df* between windows needs to be approximately constant. We therefore suggest that windows be dynamically adjusted in length to account for the temporal variability in *df*, for sliding window analyses (see Fig. 7). After ensuring minimal variability in *df* across windows, connectivity results can be probabilistically thresholded within windows and compared across time, thus minimizing spurious results related to temporal fluctuations in *df*. An important point to note here is that the high-pass filter selected during pre-processing should be sufficient to account for the shortest window size in order to avoid spurious fluctuations (Leonardi and Van De Ville, 2015).

### 4.4 Further considerations and future work

One important consideration is the choice of wavelet. Here we used the fourth-order Daubechies wavelet with filter length *L* = 8 and *M* = 4 (where *M* is the number of vanishing moments). The two properties of the wavelet despiking algorithm (denoising and probabilistic inference) each favor different wavelets. More compact support (smaller *L*) should improve denoising, but larger *L* theoretically favors better decorrelation. The final choice of wavelet should thus balance both of these factors. In the data we analyzed, we did not find that increasing *L* above 8 provided improved decorrelation, whereas use of wavelets with longer *L* did worsen denoising of some types of artifact (namely large abrupt artifacts), hence our final choice of wavelet. In practice, the findings we present in the Results section were robust to choice of wavelet, tested up to filter lengths of *L* = 16. However, application of wavelet despiking to different data may require consideration of different wavelets (a number of wavelet bases are included in the BrainWavelet Toolbox, please see Section 6 below). In general, as decorrelation across and within scales theoretically improves as a function of filter length (provided that *M* > 3), it is advisable to select the longest filter length that still allows good denoising. A further discussion on filter length and decorrelation is provided in Appendix A. Relatedly, we note that it is possible to estimate time series correlations for functional connectivity analysis in the wavelet domain, using the wavelet detail coefficients themselves. For sliding window approaches, selection of more symmetric wavelets would thus be pertinent if analysis is to be performed in the wavelet domain.

In addition, we note that the methods described in this article are general and could be combined with other analysis methods, such as the two-step thresholding procedures for connectomes described in Meskaldji et al. (2015). Wavelet despiking and the analysis methods described here have been tested on, and can also be applied to, fMRI data collected using different acquisition methods, such as short-TR, multi-band and multi-echo data, and on other biological data such as electrophysiological time series. Of note, the algorithm has proven effective at denoising up to 30 min of local field potential data sampled at 500 Hz (Donnelly et al., 2014). Finally, we note that neighboring voxel-wise *effective df* estimates could be pooled to account for spatial dependence in autocorrelation. In an analogous way to regularization of autoregressive coefficient estimation advocated for pre-whitening methodologies (Woolrich et al. (2001), based on insights by Bullmore et al. (1996)), it is possible that local regularization of *df* estimates produced by wavelet despiking may be advantageous. This could be done by local averaging of results from 1D discrete wavelet transforms or by use of higher dimensional discrete wavelet transforms, for example, as illustrated by Breakspear et al. (2004).

## 5 Conclusion

In summary, we demonstrate a new method for estimating the statistical properties of fMRI networks using wavelet despiking. This method enables estimation of *effective* degrees of freedom (*df*) by using the whitening properties of discrete wavelet transforms, to provide a spatially-variable estimate of voxel-wise *df* after artifact denoising. Using this method, we demonstrate robust Type I error controlled probabilistic inference for seed connectivity analyses and a new methodology for probabilistic interpretation of graph theoretic results.

## 6 Software

We accompany this article with a new release of the BrainWavelet Toolbox (www.brainwavelet.org) which includes code to automatically generate voxel-wise *effective* degrees of freedom (*df*) maps after wavelet despiking, for individual subjects. This version also includes tools for generating single-subject statistical maps for seed connectivity and graph theoretic analysis. The parcellation template used in this article will also be made available on this website.

## Appendix A A note on decorrelation of wavelet coefficients

The correlation between wavelet detail coefficients of the Discrete Wavelet Transform (DWT), within and between scales, is known to be small. In other words, DWTs are decorrelating. More specifically, it has been shown that the correlation between two wavelet coefficients within the same scale decays hyperbolically fast with time (Tewfik, 1992) and exponentially fast across scales (Dijkerman and Mazumdar, 1992) for a class of signals called fractional Brownian motion (fBm). These properties have also been shown for a more general wider class of 1/*f*-type signals, like fMRI time series (Wornell, 1993, Wornell, 1996). For 1/*f*-type processes, it can be shown that the correlation between any two wavelet detail coefficients is:

(13) $$\langle {\tilde{W}}_{j,t},{\tilde{W}}_{j',t'}\rangle ∼O|2^{j}t-2^{j'}t'|{}^{2\left(H-M\right)}\text{,}$$

where 〈 ⋅ 〉 represents the expected correlation, *j* is the scale, *t* is the index of the coefficient in the time plane, *H* is the Hurst exponent and *M* is the number of vanishing moments of the mother wavelet.

Provided then that *M* > 2 (and given that *H* < 1 for fBm processes), the correlation between any pair of wavelet coefficients, within or between scales, will fall as an inverse power of their separation distance. More specifically, it can be shown that for the hyperbolic and exponential decay of inter-coefficient correlation within and between scales (respectively) to hold true, → *M* > 2H + 1 (Dijkerman and Mazumdar, 1992). Decorrelating fractal processes with *H* < 1 thus requires *M* > 3. In theory, decorrelation will improve with larger *M*. However, in practice, and as described in Bullmore et al. (2001), increasing *M* does not necessarily improve decorrelation. This is because the filter length (or local support) of the wavelet, *L*, is ≥ 2*M*-1. Thus, for periodic boundary conditions, larger *L* results in a greater number of boundary coefficients and greater uncertainty in inter-coefficient correlation (Tewfik, 1992). In addition, with wavelet despiking, there is a trade-off between decorrelation and denoising. The former favors larger *M*(> 3) and the latter favors smaller *L* and thus smaller *M*. In this article, we used the fourth-order Daubechies wavelet with filter length *L* = 8 and *M* = 4 (Bullmore et al., 2001, Laird et al., 2004), which we found to denoise well while providing good decorrelation. We confirmed this by assessing the Type I error of false positive results. While we found that increasing *L* up to twice the value used in this article did not much improve the decorrelation obtained with *L* = 8, the denoising was less sensitive. In general, we suggest that the wavelet choice should be determined by the largest *L* that provides good denoising, while ensuring *M* > 3.
